# Supplementary material for: Effect of PCSK9 Inhibitors on Regulators of Lipoprotein Homeostasis, Inflammation and Coagulation
Source: Biomedicines. 2025 Jan 24;13(2):294. doi: 10.3390/biomedicines13020294 (PMC11852752; doi:10.3390/biomedicines13020294)
Supplement: Supplementary file 1 [file biomedicines-13-00294-s001.zip › biomedicines-3416608-supplementary.pdf]

Supplementary Table S1. Primer pair sequences used for the gene expression measurement.

Gene symbol	Primer label	Primer sequence (5´-3´)
SREBP1	SREBP1-F	ATCGACTACATTCGCTTTCT
	SREBP1-R	CAGATCCTTCAGAGATTTGC
LDLR	LDLR-F	AGGAGACGTGCTTGTCTGTC
	LDLR-R	CTGAGCCGTTGTCGCAGT
LIPC	LIPC-F	TGTGCAACTCTCTCGAAGCC
	LIPC-R	ATCCAGCCCTGTGATTCTCC

F – forward primer. R – reverse primer. *SREBP1* – sterol regulatory element-binding protein 1. *LDLR* – low density lipoprotein receptor. *LIPC* – hepatic lipase type C.
